# Gestational Exposure of Mice to Secondhand Cigarette Smoke Causes Bronchopulmonary Dysplasia Blocked by the Nicotinic Receptor Antagonist Mecamylamine

**DOI:** 10.1289/ehp.1306611

**Published:** 2013-06-11

**Authors:** Shashi P. Singh, Sravanthi Gundavarapu, Kevin R. Smith, Hitendra S. Chand, Ali Imran Saeed, Neerad C. Mishra, Julie Hutt, Edward G. Barrett, Matloob Husain, Kevin S. Harrod, Raymond J. Langley, Mohan L. Sopori

**Affiliations:** 1 Lovelace Respiratory Research Institute, Albuquerque, New Mexico, USA; 2 Pulmonary and Critical Care Medicine, University of New Mexico, Albuquerque, New Mexico, USA; 3 Department of Microbiology and Immunology, University of Otago, Dunedin, New Zealand

**Keywords:** angiogenesis, bronchopulmonary dysplasia, cigarette smoke, nicotinic receptors, secretory/surfactant proteins

## Abstract

Background: Cigarette smoke (CS) exposure during gestation may increase the risk of bronchopulmonary dysplasia (BPD)—a developmental lung condition primarily seen in neonates that is characterized by hypoalveolarization, decreased angiogenesis, and diminished surfactant protein production and may increase the risk of chronic obstructive pulmonary disease.

Objective: We investigated whether gestational exposure to secondhand CS (SS) induced BPD and sought to ascertain the role of nicotinic acetylcholine receptors (nAChRs) in this response.

Methods: We exposed BALB/c and C57BL/6 mice to filtered air (control) or SS throughout the gestation period or postnatally up to 10 weeks. Lungs were examined at 7 days, 10 weeks, and 8 months after birth.

Results: Gestational but not postnatal exposure to SS caused a typical BPD-like condition: suppressed angiogenesis [decreased vascular endothelial growth factor (VEGF), VEGF receptor, and CD34/CD31 (hematopoietic progenitor cell marker/endothelial cell marker)], irreversible hypoalveolarization, and significantly decreased levels of Clara cells, Clara cell secretory protein, and surfactant proteins B and C, without affecting airway ciliated cells. Importantly, concomitant exposure to SS and the nAChR antagonist mecamylamine during gestation blocked the development of BPD.

Conclusions: Gestational exposure to SS irreversibly disrupts lung development leading to a BPD-like condition with hypoalveolarization, decreased angiogenesis, and diminished lung secretory function. Nicotinic receptors are critical in the induction of gestational SS–induced BPD, and the use of nAChR antagonists during pregnancy may block CS-induced BPD.

## Introduction

Bronchopulmonary dysplasia (BPD), first described in 1967 in premature infants with respiratory distress syndrome (Northway et al. 1967), is associated with fewer and enlarged alveoli, suppressed angiogenesis, lack of or insufficient production of surfactant proteins (SPs), and lung inflammation; however, impaired alveolar development and SP deficiency remains the main pathophysiological manifestation of BPD (Allen et al. 2003). BDP is the second leading cause of mortality among the infants born before 28 weeks of gestation (Callaghan et al. 2006).

Exposure to a wide range of chemicals and environmental toxicants during pre- and perinatal life may affect the maturation and function of the respiratory system (Pinkerton and Joad 2000). The risk of cigarette smoke (CS)–associated pulmonary complications is highest during fetal and early postnatal life (DiFranza et al. 2004), yet nearly one-third of prospective mothers smoke during some stages of pregnancy (Hylkema and Blacquiere 2009). Epidemiological evidence and animal experiments suggest that parental, particularly maternal, smoking or nicotine exposure adversely affects the pulmonary health of the offspring, including a higher risk for developing allergic asthma, and airway remodeling affecting function (Rehan et al. 2009; Sekhon et al. 2001; Singh et al. 2009; Vrijheid et al. 2012). However, it remains unclear whether gestational and/or early postnatal exposure causes BPD. This is an important question because infants with BPD are at increased risk of developing chronic obstructive pulmonary disease (COPD) later in life (Ali and Greenough 2012; Broström et al. 2010; Narang 2010). In the present study, we show that gestational but not early postnatal exposure of mice to secondhand cigarette smoke (SS) suppresses alveolarization, angiogenesis, and development of Clara and goblet cells without significant lung inflammation and that the SS-induced effects on alveolar architecture are irreversible. Importantly, simultaneous exposure to the nicotinic acetylcholine receptor (nAChR) antagonist mecamylamine (MM) blocks the effects of gestational SS on lung function and alveolar architecture.

## Materials and Methods

*Animals*. Pathogen-free BALB/c or C57BL/6 mice (Frederick National Laboratory for Cancer Research at Fort Detrick, Frederick, MD, USA) were housed in shoebox-type plastic cages with hardwood chip bedding and conditioned to whole-body exposure in exposure chambers for 2 weeks before exposure to SS. The chamber temperature was maintained at 26 ± 2°C, and lights were set to a 12-hr on/off cycle. Food and water were provided *ad libitum*. All animal protocols were approved by the Institutional Animal Care and Use Committee of the Lovelace Respiratory Research Institute. Experimental animals were treated humanely and with regard for alleviation of suffering.

*CS generation and exposure*. Briefly, 70-cm^3^ puffs at the rate of 2/min were generated from 2R1 research cigarettes (Tobacco Health Research Institute, Lexington, KY, USA) by a smoking machine (Type 1300; AMESA Electronics, Geneva, Switzerland). The smoke was captured from the lit end of the cigarette with a plastic manifold placed above it. Male and female mice were exposed to whole-body SS or filtered air (FA) for 6 hr/day, 7 days/week (total particulate matter 1.52 ± 0.41 mg/m^3^) as described previously (Singh et al. 2011). This exposure is approximately equivalent to the dose of SS that a pregnant woman would receive by sitting in a smoky bar for 3 hr/day. Immediately after the birth of pups, smoke exposure was stopped; however, some gestationally FA-exposed pups also were exposed to SS until 10 weeks after birth (postnatal SS). [For additional details, see Supplemental Material, p. 3 (http://dx.doi.org/10.1289/ehp.1306611).]

*MM treatment*. Before SS or FA exposure, some breeder mice were implanted with subcutaneous mini-osmotic pumps (ALZET Osmotic Pumps, Cupertino, CA, USA) (pumping rate, 0.15 µL/hr) containing saline or MM (2.5 mg/mL), generating the FA (control), MM+FA, SS, and MM+SS groups. Animals were sacrificed at 7 days, 10 weeks, or 8 months after birth.

*Bronchoalveolar lavage fluid (BALF) collection*. Established protocols were followed to obtain BALF from 10-week-old FA (control) and SS-exposed mice (Singh et al. 2009). The right pulmonary lobe was lavaged twice with 0.8 mL sterile Ca^2+^/Mg^2+^-free phosphate-buffered saline. The vascular endothelial growth factor (VEGF) level in BALF was determined by ELISA (Biosource-Invitrogen, Carlsbad, CA, USA), according to the manufacturer’s directions. Right lungs from some animals were frozen instantaneously in liquid nitrogen and stored at –80 °C for Western blot analysis and quantitative polymerase chain reaction (qPCR) for total RNA. [For additional details, see Supplemental Material, pp. 3–4, 7–8, (http://dx.doi.org/10.1289/ehp.1306611).]

*Tissue preparation*. After sacrifice, lungs were removed, inflated and formalin-fixed at a constant hydrostatic pressure (Fritzell et al. 2009). Immediately after inflation, the trachea was ligated and the lungs were immersed in formalin overnight. [For additional details, see Supplemental Material, p. 4 (http://dx.doi.org/10.1289/ehp.1306611).]

*Hematoxylin and eosin (H&E) staining*. Standard protocols were followed for H&E staining (Singh et al. 2011; Wang et al. 2001). The slides were examined with a Nikon Eclipse E600W microscope (Nikon, Tokyo, Japan) with a digital camera.

*Morphometry*. The mean linear intercept (L_m_) was determined on H&E-stained tissue sections using a Nanozoomer Digital Pathology (NDP) slide scanner (Hamamatsu K.K. Photonics, Hamamatsu City, Japan). L_m_ was obtained by dividing the NDP-generated total length of a line drawn across the lung section (scan resolution 20×) by the total number of intercepts encountered per lung section as described previously (Knudsen et al. 2010; Thurlbeck 1967). The slides were scored for crested alveolar septa using the NDP system with Visiopharm software (Hamamatsu K.K. Photonics) in five randomly computer-selected areas of each lung slide (16,000 µm of alveolar area) of 7-day-, 10-week-, and 8-month-old mice.

*Immunohistochemical (IHC) and immunofluorescence staining*. Lung sections (5 µm) were deparaffinized and hydrated, followed by antigen retrieval (Tompkins et al. 2009). IHC and immunofluorescence were performed to visualize and quantitate the following marker-positive cells:

Surfactant protein-B–positive (SP-B^+^) (1:1,000 dilution; rabbit proSP-B antibody, catalog no. AB3430; Chemicon International, Temecula, CA, USA)
Clara cell secretory protein–positive (CCSP^+^) (Clara cells marker, 1:50,000 dilution; catalog no. 957; Vector)
β-Tubulin^+^ (ciliated cells marker, rabbit anti-β-tubulin antibody from Abcam (Cambridge, UK) followed by Alexa Fluor® 594 fluorescent dye 594–conjugated anti-rabbit antibody)
SPDEF^+^ [goblet cells marker, guinea pig anti-mouse SPDEF antibody (gift from J. Whitsett, Cincinnati Children’s Hospital, Cincinnati, OH, USA)]
CD34^+^ [a hematopoietic progenitor cell marker; anti-mouse CD34 antibody, 1:2,000 dilution, (catalog no. 119301; BioLegend, San Diego, CA, USA) followed by Alexa 555–conjugated anti-rat antibody] and/or CD31^+^ [an endothelial cell marker; rabbit anti-CD31, 1:2,000 dilution (catalog no. AB28364; Abcam) followed by DyLight 549–conjugated anti-rabbit antibody).

Proximal and distal lung airways images were acquired using CCSP- and SP-B–stained lung sections (Nikon Eclipse E600W microscope) to access SS-effect throughout the lung [for additional details, see Supplemental Material, pp. 4–8 (http://dx.doi.org/10.1289/ehp.1306611)].

Trichrome staining. Lung sections were stained with Masson’s Trichrome (Sigma-Aldrich, St. Louis, MO, USA) to reveal collagen following standard protocol [for additional details, see Supplemental Material, p. 7 (http://dx.doi.org/10.1289/ehp.1306611)].

*Quantitation of IHC-stained SP-B^+^, CCSP^+^, and ciliated epithelial cells*. The slides were scored for SP-B^+^ and CCSP^+^ cells blind using the IHC image analysis software system, NDP.Analyze (Hamamatsu K.K. Photonics) with Visiopharm software (Hoersholm, Denmark). After random selection by computer, four areas of the each lung slide were quantified using 9,000 µm of airways basal lamina. A similar approach was used for the quantitation of surface cilia stained with anti–β-tubulin antibody.

*Western blot analysis*. CD34 were quantitated by Western blotting of the lung homogenates as described previously (Singh et al. 2011). The blots were probed with anti-CD34 antibody. Antibody-bound proteins on the blot were detected using enhanced chemiluminescence on X-ray film. [For additional details, see Supplemental Material, pp. 7–8 (http://dx.doi.org/10.1289/ehp.1306611).]

*RNA isolation and qPCR*. Lung RNA was isolated as described previously (Singh et al. 2009). The lung expression of SP-B, SP-C, SP-A, CCSP, VEGF receptor 2 (VEGFR2), and glyceraldehyde 3-phosphate dehydrogenase (GAPDH) were determined by qPCR using the One-Step Real-Time PCR MasterMix containing TaqMan probes and a specific-labeled primer/probe set (Applied Biosystems).

*Data presentation and statistical analysis*. Data were analyzed using GraphPad Prism software, version 5.03 (Graphpad Software Inc., San Diego, CA, USA). We used one-way analysis of variation (ANOVA) to compare the mean between the groups, followed by the Tukey post hoc test to compare all groups at 95% confidence intervals (CIs). Student’s *t* test was used for comparison between two groups. Results are expressed as the mean ± SD. A *p*-value of ≤ 0.05 was considered statistically significant.

## Results

**Figure 1:**
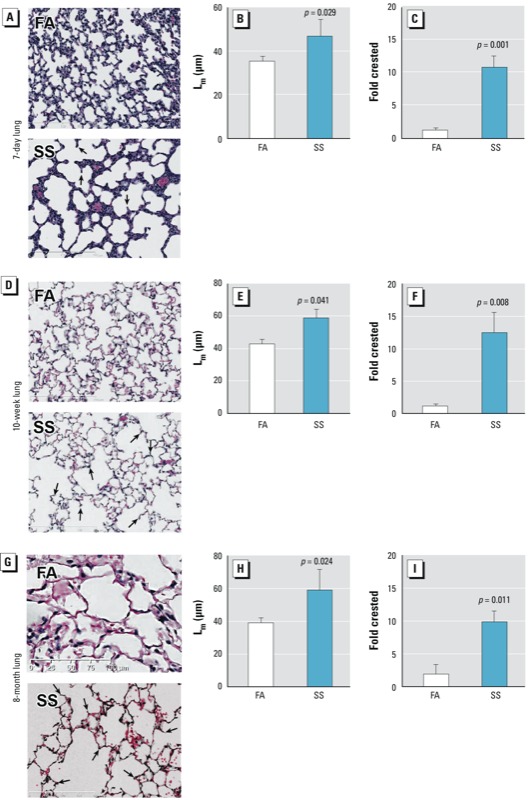
Gestational SS exposure affects normal alveolar development in mouse lung. Mice were gestationally exposed to FA or SS and evaluated at 7 days (*A–C*), 10 weeks (*D–F*), or 8 months (*G–I*) of age. Representative lung sections at 20× magnification (*A,D,G*), L_m_ (*B,E,H*), and fold change of crested alveolar septa (*C,F,I*) of lungs from mice. In *A*, *D*, and *G*, arrows indicate crested secondary septae. Data are presented as mean ± SD (*n* = 5). *p* ≤ 0.05 is statistically significant.

*Prenatal SS exposure suppresses alveolarization*. To determine the effect of prenatal SS exposure on BPD, lung sections from mice exposed gestationally to FA (control) or SS were examined microscopically at 7 days, 10 weeks, and 8 months after birth. H&E staining of lung sections suggested that gestational exposure to SS caused areas of hypoalveolarization in the lung that were seen in the early lung (7 days) as well as at 10 weeks and 8 months after the birth (Figure 1A,D,G, respectively). Changes in lung septation were quantitated by determining the L_m_ of alveoli. Compared to FA animals, the lungs from gestationally SS-exposed animals showed similar increases in L_m_ values (approximately 25%) at 7 days, 10 weeks, and 8 months after birth (Figure 1B,E,H, respectively). Moreover, histopathology of the alveolar septae showed that instead of the elongated secondary septa seen in control lungs, SS-exposed lungs had an increased presence of crested secondary septae, indicating incomplete formation of secondary alveolar septa (Figure 1A,D,G)—a feature also seen in human BPD (Ahlfeld and Conway 2012). In lung sections, these crested structures were counted and fold changes over FA-exposed animals were plotted, showing significant increases in secondary crested structures in gestationally SS-exposed animals at 7 days, 10 weeks, and 8 months after birth (Figure 1C,F,I, respectively). On the other hand, postnatal exposure to SS [postnatal day (PD) 1 through 10 weeks] did not cause a significant change in alveolarization, and L_m_ values were comparable to those of control animals [see Supplemental Material, Figure S1A (http://dx.doi.org/10.1289/ehp.1306611)]. These results indicate that *in utero* but not early postnatal SS exposure impairs alveolar septation, leading to irreversible hypoalveolarization. To ascertain whether BALB/c mice were uniquely susceptible to gestational SS, we also exposed C57BL/6 mice to gestational SS. Results from SS-exposed PD7 lungs from C57BL/6 mice (see Supplemental Material, Figure S1B) showed morphometric changes similar to those observed in BALB/c mice, thus indicating that both BALB/c and C57BL/6 mice develop impaired alveolarization in response to gestational SS.

In addition to impaired postnatal lung growth, BPD may cause variable interstitial fibrosis (Kinsella et al. 2006); however, collagen structure (identified by trichrome staining) did not show any significant difference between FA- and SS-exposed 10-week-old lungs [see Supplemental Material, Figure S2 (http://dx.doi.org/10.1289/ehp.1306611)]. Moreover, there was no indication of leukocytic infiltration in gestationally SS-exposed lungs (not shown). These results suggest that the SS-induced hypoalveolarization is not associated with significant lung fibrosis or inflammation.

**Figure 2:**
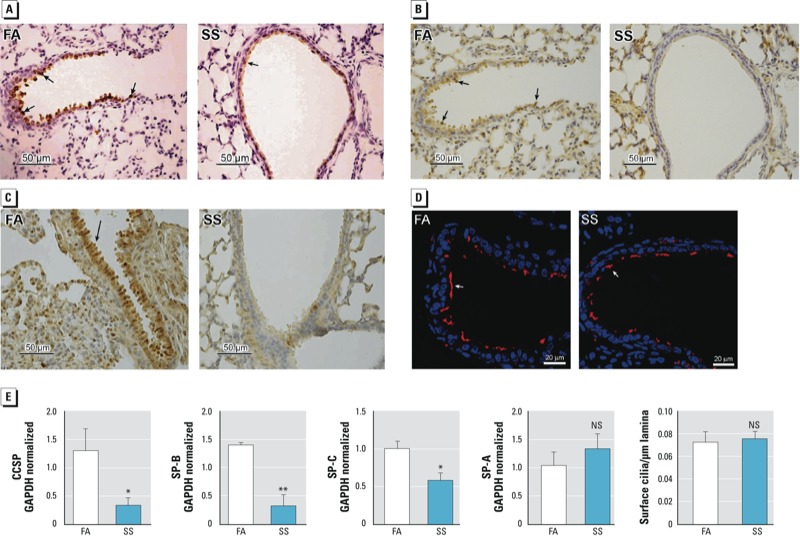
Gestational SS exposure impairs the development of Clara and goblet cells but not ciliated cells in mouse lung. Mice were gestationally exposed to FA or SS and evaluated at 7 days of age. Lung sections (40× magnification) stained with (*A*) anti-CCSP antibody, (*B*) anti–SP-B antibody, (*C*) anti-SPDEF antibody, and (*D*) anti-β-tubulin antibody for ciliated cells (immunofluorescence image). (*E*) qPCR analysis for CCSP, SP-B, SP-C, SP-A, and surface cilia per micrometer of basal lamia. NS, not significant. Arrows show stained cells in the airways. Data are presented as mean ± SD (n = 3–5). *p ≤ 0.05. **p ≤ 0.01.

*Gestational SS exposure impairs the development of Clara and goblet cells but not ciliated epithelium cells*. Down-regulation of secretory proteins contributes to the pathology of several airway diseases, including BPD, and many premature infants are given a mixture of surfactant proteins to improve lung function (Kinsella et al. 2006; Merrill et al. 2011). To determine whether gestational SS broadly affected the airway secretory functions, we examined airway Clara and goblet cells in FA- and SS-exposed mouse lung sections at PD7 by staining for CCSP and SP-B (Clara cell markers) and SPDEF (goblet cell marker). As seen in Figure 2A (CCSP), Figure 2B (SP-B), and Figure 2C (SPDEF), the presence of Clara and goblet cells were significantly reduced in the SS-exposed lung. However, ciliated (β-tubulin^+^) cells (Figure 2D) were not significantly changed by SS exposure in PD7 lung. qPCR analysis (Figure 2E) also showed decreased mRNA expression of CCSP, SP-B, and SP-C. On the other hand, the mRNA expression of SP-A and the number of β-tubulin+ cells per micrometer of basal lamina (Figure 2E) did not show any significant changes between gestationally FA- and SS-exposed lungs. These results indicate that prenatal exposure to SS impairs the development of Clara and goblet cells, which contributes to the reduced production of surfactant proteins and airway mucus in gestationally SS-exposed lungs.

To determine whether gestational SS affected the airway secretory cells uniformly, the distribution of CCSP and SP-B in the airways was assessed by IHC staining in the proximal and distal airways. Results indicated that both proximal and distal airways were affected similarly by gestational SS [see Supplemental Material, Figures S3 and S4 (http://dx.doi.org/10.1289/ehp.1306611)].

**Figure 3:**
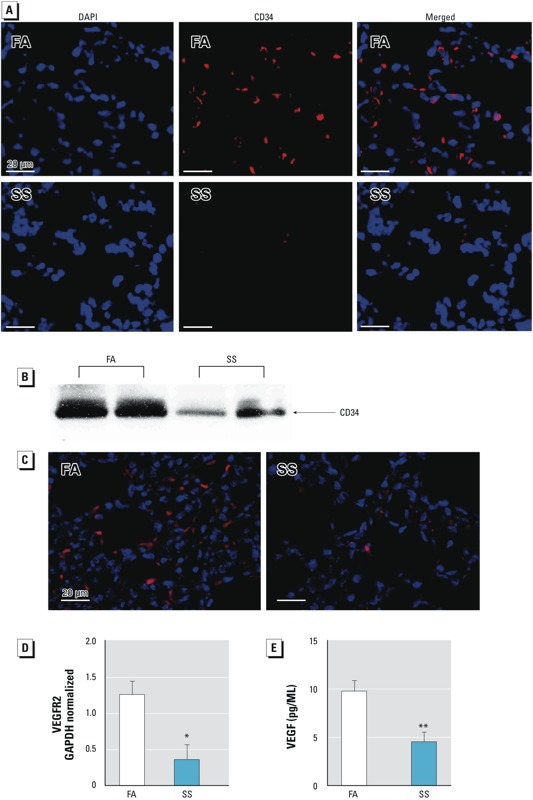
Gestational SS exposure inhibits angiogenesis. Mice were gestationally exposed to FA or SS. (*A*) Immunofluorescence images (400× magnification) of representative lung sections from 7-day-old mice stained with anti-CD34/anti-rat conjugated Alexa 555. Nuclei are indicated by blue fluorescence and CD34 by red fluorescence. (*B*) Western blot of protein from 70 μg lung homogenate from 7‑day-old mice. (*C*) Representative immunofluorescence image of lung from 7‑day-old mice stained with anti-CD31 (PECAM‑1). (*D*) VEGFR2 in lung from 7‑day-old mice determined by qPCR. (*E*) VEGF level in BALF in lung from 10-week-old mice determined by ELISA. Data are presented as mean ± SD (*n* = 5). *p ≤ 0.05. **p ≤ 0.01.

*Gestational SS impairs airway angiogenesis*. Angiogenesis is a tightly regulated physiological process that occurs during embryogenesis (Breier 2000) and plays a vital role in the development of the lung and airways (McCullagh et al. 2010), and new alveolar septa formation is closely associated with microvascular maturation (Burri 2006). We evaluated lung vascularization by immunofluorescence by staining for CD34, a transmembrane glycoprotein expressed primarily by endothelial cells and strongly present in alveolar wall capillaries (Pusztaszeri et al. 2006). Tissue sections were also stained for cell nuclei with DAPI. Compared to controls (FA), the SS-exposed lungs at PD7 exhibited much weaker immunofluorescence (Figure 3A) for CD34, indicating a decreased number of endothelial cells in gestationally SS-exposed lungs. Western blots of the lung extracts from FA- and SS-exposed animals also showed that SS-exposed lungs had significantly reduced levels of CD34 immunoreactive protein (Figure 3B). Decreased angiogenesis in SS-exposed lungs was further confirmed by immunofluorescence for CD31 (Figure 3C), a major constituent of endothelial cell intercellular junctions (Muller et al. 1989) that is absent on nonvascular cells such as epithelium, fibroblasts, and muscle cells (Newman et al. 1990). These results indicate that gestational SS exposure strongly suppresses/impairs vascular development in the lung.

Coordinated and timely release of angiogenic growth factors from respiratory epithelial cells promotes normal alveolar development (Thebaud et al. 2005). Although multiple factors affect angiogenesis, VEGF plays an important role in postnatal lung alveolar development as well as in the maintenance of alveolar structures in the adult lung (Carmeliet et al. 1996; Kasahara et al. 2000; Ng et al. 2001; Ruhrberg 2003; Zhao et al. 2005). The expression of VEGFRs increases during lung development, and most of the VEGF effects are mediated through VEGFR2 (Kalinichenko et al. 2001; Ng et al. 2001). We determined the expression of VEGFR2 by qPCR in the PD7 lung from FA- and SS-exposed mice. VEGFR2 expression was significantly reduced in the SS-exposed animals (Figure 3D). Moreover, the concentration of VEGF in BALF from SS-exposed mouse lungs at 10 weeks after birth was significantly lower than in BALF from control lungs (Figure 3E). These results suggest that gestational exposure to SS causes angiogenic defects in the developing lung, and the decreased expression of VEGF and its main receptor VEGFR2 are likely to contribute to the defective angiogenesis of the lung in gestationally SS-exposed animals.

**Figure 4:**
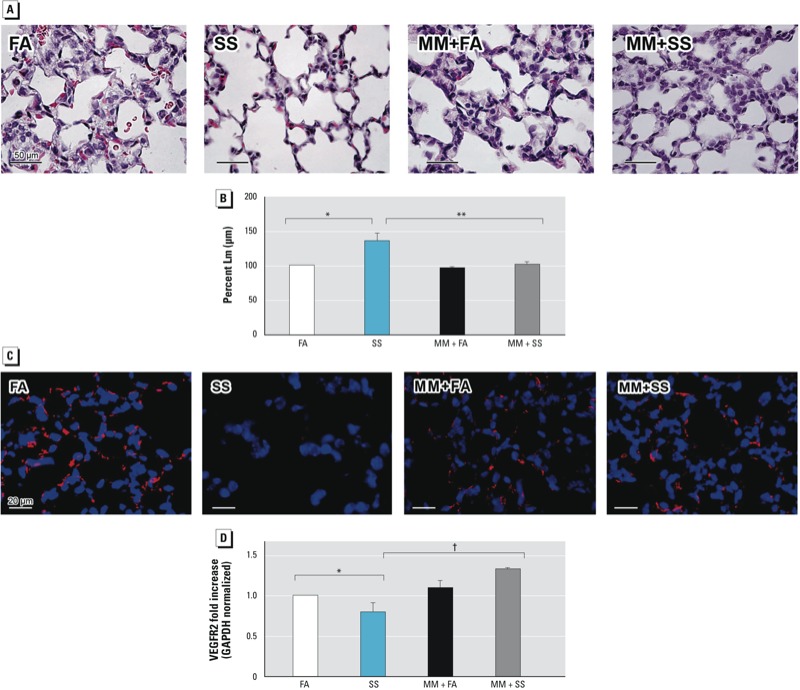
Gestational SS-induced effects on alveolarization and angeiogenesis are blocked by MM. Mice were gestationally exposed to FA or SS with or without MM exposure and evaluated at 7 days of age. (*A*) Representative lung sections (40× magnification: stained with H&E). (*B*) Percent L_m_. (*C*) Representative immunofluorescence images of lung sections stained with anti-CD31 (PECAM-1)/anti-rabbit DyLight 549. (*D*) qPCR analysis of VEGFR2. Data are presented as mean ± SD (*n* = 5). *p ≤ 0.05, compared with FA. **p ≤ 0.01, and ^†^*p* ≤ 0.001, compared with SS.

**Figure 5:**
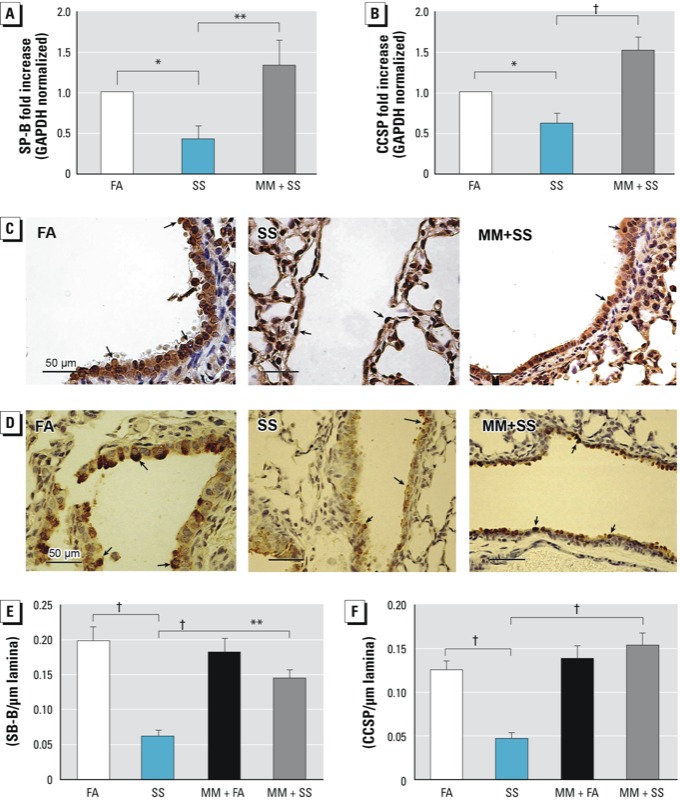
Gestational SS-induced effect on Clara cells is blocked by MM. Mice were gestationally exposed to FA or SS with or without MM exposure and evaluated at 7 days of age. qPCR analysis of SP-B (*A*) and CCSP (*B*) in lung. Representative IHC image (40× magnification) of lung stained for SP-B (*C*) and CCSP (*D*); arrows indicate positive cells in the lung airways. SP-B^+^ (*E*) and CCSP^+^ (*F*) cells per micrometer of basal lamina. Data are presented as mean ± SD (*n* = 5). *p ≤ 0.05. **p ≤ 0.01. ^†^*p* ≤ 0.001.

*MM blocks SS-induced effects on lung pathology.* Nicotine is the major component of SS. Therefore, it was possible that the SS-induced BPD-like condition was regulated by nAChRs, and blocking these receptors would prevent the gestational SS-induced injury to the lung. We observed that while MM (an nAChR antagonist) treatment during gestational period alone did not significantly affect alveolarization and L_m_ of PD7 lung, it blocked the effects of gestational SS on alveolar septation (Figure 4A) and L_m_ values (Figure 4B). Furthermore, immunofluorescence staining for CD31 (Figure 4C) of the lung section and qPCR analysis for VEGFR2 (Figure 4D) indicated that pretreatment with MM also normalized lung vascularization (CD31 expression) and the VEGFR2 level in the PD7 lung. Similarly, qPCR analysis indicated that MM restored the levels of SP-B (Figure 5A) and CCSP (Figure 5B) and also suppressed the SS-induced reduction in the airway SP-B staining (Figure 5C) and CCSP staining (Figure 5D). MM countered the effects of SS on SP-B^+^ cells (Figure 5E) and CCSP^+^ cells (Figure 5F). Moreover, MM restored CCSP^+^ and SP-B^+^ cells throughout the airways [see Supplemental Material, Figures S3 and S4 (http://dx.doi.org/10.1289/ehp.1306611)]. Thus, MM treatment essentially blocks the inhibitory effects of gestational SS on alveolarization, vascularization, and secretory/surfactant protein production, indicating a critical role of nAChRs in the development of SS-induced BPD.

## Discussion

Increasing evidence suggests that *in utero* exposure to environmental toxins, including polycyclic aromatic hydrocarbons and CS/nicotine affect lung development and function (Perera et al. 2009; Rehan et al. 2009; Sekhon et al. 2001; Singh et al. 2011; Vrijheid et al. 2012). In addition, in rats maternal exposure to nicotine during gestation and lactation produced oxidative stress–related lung impairment, including microscopic emphysema during adult life (Maritz and Rayise 2011). In humans, intrauterine exposure to CS has been deemed a risk factor for BPD (Antonucci et al. 2004); however, to our knowledge, this has remained unconfirmed. BPD is a disease whose etiology has not been fully established. Here we present evidence that gestational, but not early postnatal exposure of BALB/c and C57BL/6 mice to SS causes BPD and the PD7 lung exhibit hypoalveolarization and significant increases in L_m_ (≥⊇23%) comparable to some patients with COPD/emphysema (Jacob et al. 2009).

Chronic exposure to CS is the most important cause of COPD/emphysema in humans, and the loss of alveolar surface is associated with increased concentrations of metalloproteinases (MMP) (Mocchegiani et al. 2011). MMP-12 is implicated in CS-induced emphysema (Belvisi and Bottomley 2003); however, MMP-12 was not elevated in SS-exposed mice (not shown), suggesting that, although classical emphysema is progressive and results from dissolution of preformed alveolar septae, the SS-induced BPD results from impaired formation of secondary alveolar septae and, in the absence of additional lung insults, is not progressive. This inference is further supported by the histopathology of SS-exposed PD7 lung showing the increased presence of crested secondary septa (i.e., incomplete formation of secondary alveolar septa). These results suggest that gestational SS-induced BPD results primarily from impaired alveolar maturation (Albertine et al. 2010).

Interaction between epithelial and vascular compartments is critical in alveologenesis, and coordinated and timely release of angiogenic growth factors from respiratory epithelial cells promotes normal alveolar development (Thebaud et al. 2005). The development of fewer alveoli and microvessels is a quintessential feature of neonatal chronic lung disease (D’Angio and Maniscalco 2004) and pulmonary endothelium is closely apposed to the developing epithelium (Ng et al. 2001). VEGF is the most potent mediator of angiogenesis (Carmeliet et al. 1996; Ng et al. 2001), and it plays a significant role in the pathophysiology of common respiratory disorders, including acute lung injury, asthma, COPD, pulmonary fibrosis, and lung cancer (Papaioannou et al. 2006). The angiogenic effects of VEGF are primarily mediated through VEGFR2 (Kalinichenko et al. 2001; Ng et al. 2001). Consistent with the critical role of VEGF and VEGFR2 in BPD, human infants who die of BPD have little or no VEGF in their lung epithelium. BPD is also associated with decreased levels of angiogenic progenitor cells in cord blood (Baker et al. 2012) and reduction of VEGFRs in pulmonary vasculature (Thebaud et al. 2005). CD34 is present on endothelial cells, and is involved in leukocyte adhesion and endothelial cell migration during angiogenesis (Pusztaszeri et al. 2006). The expression of CD34 in the PD7 SS-exposed lung was severely reduced. Because CD34 is also present on progenitor hematopoietic cells (Civin et al. 1984), to ensure that SS affected the vascular endothelial cells, we also determined the expression of CD31—a major constituent of the endothelial cell intercellular junction (Muller et al. 1989). As with CD34, the lung expression of CD31 was much lower in gestationally SS-exposed animals than FA controls. Similarly, the lung expression of other parameters of angiogenesis, such as VEGF and VEGFR2 were significantly decreased in the 10-week SS-exposed lungs. Similarly, in preterm children with BPD, VEGF levels remain significantly lower than preterm children without BPD (Meller and Bhandari 2012). Decreased expression of VEGFR2 was also seen by microarray analysis of carotid arteries in monkeys exposed during gestation and early postnatal life to environmental tobacco smoke (Meller and Bhandari 2012). Together, these results suggest that decreased alveolarization in gestationally SS-exposed lung may reflect the reduction in angiogenesis that affects lung vascularization.

Another characteristic of BPD is the decreased production of CCSP and SP-B (Meller and Bhandari 2012). CCSP modulates immune responses, reduces lung injury in animal models, up-regulates the expression of surfactant proteins and VEGF in the lung, and ameliorates BPD (Ramsay et al. 2001). SP-B lowers surface tension and prevents atelectasis and protects epithelial cells (Abdel-Latif and Osborn 2011), and its administration may improve lung functions in BPD (Logan and Moya 2009; Merrill et al. 2011). The expression of CCSP, SP-B, and SP-C is significantly down-regulated in the gestationally SS-exposed PD7 lungs. However, the expression of SP-A surfactant protein was comparable in gestationally FA- and SS-exposed animals. This disparity between the expression of SP-A and other surfactant proteins in SS-induced BPD is not clear; however, in the lung SP-A is also made by airway submucosal gland cells (Saitoh et al. 1998.)

There are three major epithelial cell types in the airways: ciliated cells, Clara cells, and goblet cells. Ciliated epithelium participates in the mucociliary clearance and expresses β-tubulin on the surface (Tompkins et al. 2009), whereas Clara cells and goblet cells express CCSP and SPDEF, respectively. IHC analysis clearly indicated that gestational SS did not significantly affect ciliated (β-tubulin^+^) cells, but a drastic reduction was noted in CCSP^+^ and SPDEF^+^ cells in the airways.

Thus, the severe lack of secretory proteins in SS-exposed lungs may reflect impaired or delayed development of these cells in the SS-exposed lung. Indeed, impaired airway secretory function such as mucus formation (Singh et al. 2011) and CCSP/SP-B was evident even at 10 weeks after the birth of gestationally SS-exposed animals [see Supplemental Material, Figure S5A,B (http://dx.doi.org/10.1289/ehp.1306611)]. Decreased presence of CCSP^+^ cells was observed both in proximal and distal areas of the airways, suggesting the effects of gestational SS on maturation and differentiation of type II epithelial cells. Additionally, CCSP stimulates VEGF and alveologenesis (Abdel-Latif and Osborn 2011; Londhe et al. 2011), and the decreased expression of VEGF may contribute to impaired angiogenesis and alveologenesis in SS-exposed lungs.

SS contains many toxic chemicals, including nicotine, and nAChRs are present on many non-neuronal cell types, including endothelial (Cooke and Ghebremariam 2008) and lung epithelial cells (Gundavarapu et al. 2012). Thus, it is possible that SS affects lung alveolarization, angiogenesis, and surfactant proteins through nAChRs. Moreover, in rats nicotine treatment during gestation was shown to affect some aspects of lung development (Maritz and Rayise 2011). Treatment with the nAChR antagonist MM during the gestational period ameliorated the effects of gestational SS on alveolarization, angiogenesis, and airway secretory function. These results suggest that nAChRs are critical in SS-induced BPD. A surprising observation was that the *in utero* SS effects on some lung parameters were opposite of those observed in adult mice after CS/nicotine exposure. For example, exposure of adult mice to CS/nicotine suppresses allergic responses (Mishra et al. 2008), but prenatal exposure to SS strongly exacerbates allergen-induced atopy and T-helper 2 (Th2) cell polarization (Singh et al. 2011). Similarly, unlike its anti-angiogenic effects during gestational period, nicotine stimulates neovascularization (Cooke and Ghebremariam 2008) and airways mucus formation (Gundavarapu et al. 2012; Singh et al. 2011). A potential explanation is that long-term exposure to low levels of CS/nicotine may promote desensitization or loss of nAChRs, and these receptors may be important in regulating lung growth. Although currently we have no direct evidence to support this possibility, recent papers suggest that whereas short-term exposure to nicotine may promote angiogenesis and VEGF production, long-term exposures to nicotine may impair cholinergic angiogenesis and impair capillary sprouting (Konishi et al. 2010).

Although maternal smoking has been considered a risk factor for BPD in children (Antonucci et al. 2004), mice might be particularly sensitive to pro-BPD effects of SS. Alternatively, unlike humans, experimental mice are inbred and the presence of susceptibility factors on both alleles might make them more susceptible to CS-induced BPD. Nonetheless, this excessive susceptibility might make an excellent model to study the mechanism of BPD. Interestingly, postnatal SS had very little effect on the development of BPD. In view of recent human data suggesting that exposure to CS during the first trimester is sufficient to increase the risk of asthma in children (Vrijheid et al. 2012), CS-induced changes during early embryogenesis might be critical for BPD. Overall, the present study shows that exposure to environmental tobacco smoke during gestation interferes with alveolarization and promotes a BPD-like syndrome. These effects of SS are mediated through nAChRs, and antagonists of nAChRs may have therapeutic value in blocking the effects of CS/nicotine on fetal lung development.

## Supplemental Material

(5.1 MB) PDF

## References

[r1] Abdel-Latif ME Osborn DA 2011 Intratracheal Clara cell secretory protein (CCSP) administration in preterm infants with or at risk of respiratory distress syndrome. Cochrane Database Syst Rev (5 CD008308; 10.1002/14651858.CD008308.pub2 [Online 11 May 2011] 21563168 PMC6464311

[r2] Ahlfeld SK, Conway SJ (2012). Aberrant signaling pathways of the lung mesenchyme and their contributions to the pathogenesis of bronchopulmonary dysplasia.. Birth Defects Res A Clin Mol Teratol.

[r3] Albertine KH, Dahl MJ, Gonzales LW, Wang ZM, Metcalfe D, Hyde DM (2010). Chronic lung disease in preterm lambs: effect of daily vitamin A treatment on alveolarization.. Am J Physiol Lung Cell Mol Physiol.

[r4] Ali K, Greenough A (2012). Long-term respiratory outcome of babies born prematurely.. Ther Adv Respir Dis.

[r5] Allen J, Zwerdling R, Ehrenkranz R, Gaultier C, Geggel R, Greenough A (2003). Statement on the care of the child with chronic lung disease of infancy and childhood.. Am J Respir Crit Care Med.

[r6] Antonucci R, Contu P, Porcella A, Atzeni C, Chiappe S (2004). Intrauterine smoke exposure: a new risk factor for bronchopulmonary dysplasia?. J Perinat Med.

[r7] Baker CD, Balasubramaniam V, Mourani PM, Sontag MK, Black CP, Ryan SL (2012). Cord blood angiogenic progenitor cells are decreased in bronchopulmonary dysplasia.. Eur Respir J.

[r8] Belvisi MG, Bottomley KM (2003). The role of matrix metalloproteinases (MMPs) in the pathophysiology of chronic obstructive pulmonary disease (COPD): a therapeutic role for inhibitors of MMPs?. Inflamm Res.

[r9] Breier G (2000). Functions of the VEGF/VEGF receptor system in the vascular system.. Semin Thromb Hemost.

[r10] Broström EB, Thunqvist P, Adenfelt G, Borling E, Katz-Salamon M (2010). Obstructive lung disease in children with mild to severe BPD.. Respir Med.

[r11] Burri PH (2006). Structural aspects of postnatal lung development—alveolar formation and growth.. Biol Neonate.

[r12] Callaghan WM, MacDorman MF, Rasmussen SA, Qin C, Lackritz EM (2006). The contribution of preterm birth to infant mortality rates in the United States.. Pediatrics.

[r13] Carmeliet P, Ferreira V, Breier G, Pollefeyt S, Kieckens L, Gertsenstein M (1996). Abnormal blood vessel development and lethality in embryos lacking a single VEGF allele.. Nature.

[r14] Civin CI, Strauss LC, Brovall C, Fackler MJ, Schwartz JF, Shaper JH (1984). Antigenic analysis of hematopoiesis. III. A hematopoietic progenitor cell surface antigen defined by a monoclonal antibody raised against KG-1a cells.. J Immunol.

[r15] Cooke JP, Ghebremariam YT (2008). Endothelial nicotinic acetylcholine receptors and angiogenesis.. Trends Cardiovasc Med.

[r16] D’Angio CT, Maniscalco WM (2004). Bronchopulmonary dysplasia in preterm infants: pathophysiology and management strategies.. Paediatr Drugs.

[r17] DiFranza JR, Aligne CA, Weitzman M (2004). Prenatal and postnatal environmental tobacco smoke exposure and children’s health.. Pediatrics.

[r18] Fritzell JA, Mao Q, Gundavarapu S, Pasquariello T, Aliotta JM, Ayala A (2009). Fate and effects of adult bone marrow cells in lungs of normoxic and hyperoxic newborn mice.. Am J Respir Cell Mol Biol.

[r19] Gundavarapu S, Wilder JA, Mishra NC, Rir-Sima-Ah J, Langley RJ, Singh SP (2012). Role of nicotinic receptors and acetylcholine in mucous cell metaplasia, hyperplasia, and airway mucus formation *in vitro* and *in vivo*.. J Allergy Clin Immunol.

[r20] Hylkema MN, Blacquiere MJ (2009). Intrauterine effects of maternal smoking on sensitization, asthma, and chronic obstructive pulmonary disease.. Proc Am Thorac Soc.

[r21] Jacob RE Carson JP Gideon KM Amidan BG Smith CL Lee KM 2009 Comparison of two quantitative methods of discerning airspace enlargement in smoke-exposed mice. PloS One 4 8 e6670; 10.1371/journal.pone.0006670 [Online 18 August 2009] 19688093 PMC2722737

[r22] Kalinichenko VV, Lim L, Stolz DB, Shin B, Rausa FM, Clark J (2001). Defects in pulmonary vasculature and perinatal lung hemorrhage in mice heterozygous null for the *Forkhead Box f1* transcription factor.. Dev Biol.

[r23] Kasahara Y, Tuder RM, Taraseviciene-Stewart L, Le Cras TD, Abman S, Hirth PK (2000). Inhibition of VEGF receptors causes lung cell apoptosis and emphysema.. J Clin Invest.

[r24] Kinsella JP, Greenough A, Abman SH (2006). Bronchopulmonary dysplasia.. Lancet.

[r25] Knudsen L, Weibel ER, Gundersen HJ, Weinstein FV, Ochs M (2010). Assessment of air space size characteristics by intercept (chord) measurement: an accurate and efficient stereological approach.. J Appl Physiol.

[r26] Konishi H, Wu J, Cooke JP (2010). Chronic exposure to nicotine impairs cholinergic angiogenesis.. Vascular Med.

[r27] Logan JW, Moya FR (2009). Animal-derived surfactants for the treatment and prevention of neonatal respiratory distress syndrome: summary of clinical trials.. Ther Clin Risk Manag.

[r28] Londhe VA, Maisonet TM, Lopez B, Jeng JM, Xiao J, Li C (2011). Conditional deletion of epithelial IKKβ impairs alveolar formation through apoptosis and decreased VEGF expression during early mouse lung morphogenesis.. Respir Res.

[r29] Maritz GS, Rayise SS (2011). Effect of maternal nicotine exposure on neonatal rat lung development: protective effect of maternal ascorbic acid supplementation.. Exp Lung Res.

[r30] McCullagh A, Rosenthal M, Wanner A, Hurtado A, Padley S, Bush A (2010). The bronchial circulation—worth a closer look: a review of the relationship between the bronchial vasculature and airway inflammation.. Pediatr Pulmonol.

[r31] Meller S, Bhandari V (2012). VEGF levels in humans and animal models with RDS and BPD: temporal relationships.. Exp Lung Res.

[r32] Merrill JD, Ballard PL, Courtney SE, Durand DJ, Hamvas A, Hibbs AM (2011). Pilot trial of late booster doses of surfactant for ventilated premature infants.. J Perinatol.

[r33] Mishra NC, Rir-Sima-Ah J, Langley RJ, Singh SP, Pena-Philippides JC, Koga T (2008). Nicotine primarily suppresses lung Th2 but not goblet cell and muscle cell responses to allergens.. J Immunol.

[r34] Mocchegiani E, Giacconi R, Costarelli L (2011). Metalloproteases/anti-metalloproteases imbalance in chronic obstructive pulmonary disease: genetic factors and treatment implications.. Curr Opin Pulm Med.

[r35] Muller WA, Ratti CM, McDonnell SL, Cohn ZA (1989). A human endothelial cell-restricted, externally disposed plasmalemmal protein enriched in intercellular junctions.. J Exp Med..

[r36] Narang I (2010). Review series: What goes around, comes around: childhood influences on later lung health? Long-term follow-up of infants with lung disease of prematurity.. Chron Respir Dis.

[r37] Newman PJ, Berndt MC, Gorski J, White GC, Lyman S, Paddock C (1990). PECAM-1 (CD31) cloning and relation to adhesion molecules of the immunoglobulin gene superfamily.. Science.

[r38] Ng YS, Rohan R, Sunday ME, Demello DE, D’Amore PA (2001). Differential expression of VEGF isoforms in mouse during development and in the adult.. Dev Dyn.

[r39] Northway WH, Rosan RC, Porter DY (1967). Pulmonary disease following respirator therapy of hyaline-membrane disease. Bronchopulmonary dysplasia.. N Engl J Med.

[r40] Papaioannou AI, Kostikas K, Kollia P, Gourgoulianis KI (2006). Clinical implications for vascular endothelial growth factor in the lung: friend or foe?. Respir Res.

[r41] Perera F Tang WY Herbstman J Tang D Levin L Miller R 2009 Relation of DNA methylation of 5’-CpG island of *ACSL3* to transplacental exposure to airborne polycyclic aromatic hydrocarbons and childhood asthma. PloS One 4 2 e4488; 10.1371/journal.pone.0004488 [Online 16 February 2009] 19221603 PMC2637989

[r42] Pinkerton KE, Joad JP (2000). The mammalian respiratory system and critical windows of exposure for children’s health.. Environ Health Perspect.

[r43] Pusztaszeri MP, Seelentag W, Bosman FT (2006). Immunohistochemical expression of endothelial markers CD31, CD34, von Willebrand factor, and Fli-1 in normal human tissues.. J Histochem Cytochem.

[r44] Ramsay PL, DeMayo FJ, Hegemier SE, Wearden ME, Smith CV, Welty SE (2001). Clara cell secretory protein oxidation and expression in premature infants who develop bronchopulmonary dysplasia.. Am J Respir Crit Care Med.

[r45] Rehan VK, Asotra K, Torday JS (2009). The effects of smoking on the developing lung: insights from a biologic model for lung development, homeostasis, and repair.. Lung.

[r46] Ruhrberg C (2003). Growing and shaping the vascular tree: multiple roles for VEGF.. Bioessays.

[r47] Saitoh H, Okayama H, Shimura S, Fushimi T, Masuda T, Shirato K (1998). Surfactant protein A2 gene expression by human airway submucosal gland cells.. Am J Respir Cell Mol Biol.

[r48] Sekhon HS, Keller JA, Benowitz NL, Spindel ER (2001). Prenatal nicotine exposure alters pulmonary function in newborn rhesus monkeys.. Am J Respir Crit Care Med.

[r49] Singh SP, Gundavarapu S, Pena-Philippides JC, Rir-Sima-Ah J, Mishra NC, Wilder JA (2011). Prenatal secondhand cigarette smoke promotes Th2 polarization and impairs goblet cell differentiation and airway mucus formation.. J Immunol.

[r50] Singh SP, Mishra NC, Rir-Sima-Ah J, Campen M, Kurup V, Razani-Boroujerdi S (2009). Maternal exposure to secondhand cigarette smoke primes the lung for induction of phosphodiesterase-4D5 isozyme and exacerbated Th2 responses: rolipram attenuates the airway hyperreactivity and muscarinic receptor expression but not lung inflammation and atopy.. J Immunol.

[r51] Thebaud B, Ladha F, Michelakis ED, Sawicka M, Thurston G, Eaton F (2005). Vascular endothelial growth factor gene therapy increases survival, promotes lung angiogenesis, and prevents alveolar damage in hyperoxia-induced lung injury: evidence that angiogenesis participates in alveolarization.. Circulation.

[r52] Thurlbeck WM (1967). Measurement of pulmonary emphesema.. Am Rev Respir Dis.

[r53] Tompkins DH Besnard V Lange AW Wert SE Keiser AR Smith AN 2009 Sox2 is required for maintenance and differentiation of bronchiolar Clara, ciliated, and goblet cells. PloS One 4 12 e8248; 10.1371/journal.pone.0008248 [Online 14 December 2009] 20011520 PMC2788414

[r54] Vrijheid M, Casas M, Bergstrom A, Carmichael A, Cordier S, Eggesbo M (2012). European birth cohorts for environmental health research.. Environ Health Perspect.

[r55] Wang SZ, Rosenberger CL, Espindola TM, Barrett EG, Tesfaigzi Y, Bice DE (2001). CCSP modulates airway dysfunction and host responses in an Ova-challenged mouse model.. Am J Physiol Lung Cell Mol Physiol.

[r56] Zhao L, Wang K, Ferrara N, Vu TH (2005). Vascular endothelial growth factor co-ordinates proper development of lung epithelium and vasculature.. Mech Dev.

