# Supplementary material for: Gestational Exposure of Mice to Secondhand Cigarette Smoke Causes Bronchopulmonary Dysplasia Blocked by the Nicotinic Receptor Antagonist Mecamylamine
Source: Environ Health Perspect. 2013 Jun 11;121(8):957–64. doi: 10.1289/ehp.1306611 (PMC3734504; doi:10.1289/ehp.1306611)
Supplement: (5.1 MB) PDF [file ehp.1306611.s001.pdf]

Supplemental Material

Gestational Exposure of Mice to Secondhand Cigarette Smoke Causes Bronchopulmonary Dysplasia Blocked by the Nicotinic Receptor Antagonist Mecamylamine

Shashi P. Singh, Sravanthi Gundavarapu, Kevin R. Smith, Hitendra S. Chand, Ali Imran Saeed, Neerad C. Mishra, Julie Hutt, Edward G. Barrett, Matloob Husain, Kevin S. Harrod, Raymond J. Langley, and Mohan L Sopori

Corresponding Author:

Mohan Sopori, Ph.D.

Immunology Division, Lovelace Respiratory Research Institute

Albuquerque, NM 87108

Tel: (505) 348-9440

Fax: (505) 348-4986

Email: msopori@lrri.org

CONTENTS | Page

Materials and Methods	3
Antibodies and reagents	3
Cigarette smoke generation and exposure	3
Bronchoalveolar lavage fluid (BALF) collection and VEGF analysis	3
Tissue preparation, histochemistry, and immunostaining	4
Immunohistochemical (IHC) staining for surfactant protein-B (SP-B)	4
SPDEF staining	5
IHC staining for Clara cells	6
Immunofluorescence staining of epithelial surface cilia	6
CD34 and CD31 Immunofluorescence staining	7
Trichrome Staining	7
Western blot analysis	7
Supplemental Material Figure S1	9
Supplemental Material Figure S2	10
Supplemental Material Figure S3	11
Supplemental Material Figure S4	12
Supplemental Material Figure S5	13
References	14

MATERIALS AND METHODS

Antibodies and reagents

Unless stated otherwise, all the reagents used in these experiments were purchased from Sigma Chemical Co. (St. Louis, MO).

Cigarette smoke generation and exposure

Mice were exposed to whole-body secondhand cigarette smoke (SS, the smoke released from the burning end of a cigarette) or fresh air (FA) for 6 hours/day, 7 days/week as described (Singh et al. 2003). Adult (3−4 month old) male and female mice were separately acclimatized either to SS or FA for 2 weeks, and then paired for mating under the same exposure conditions. After ensuring pregnancy by vaginal smear, pregnant mice were separated, housed singly in plastic cages, and continued to receive SS or FA until the pups were born. Immediately after the birth of pups some mice from SS were placed in FA chamber (gestational SS-exposure only). Conversely, some mice from FA group were exposed to SS up to 10 wk after the birth (postnatal SS-exposure only). Mice were sacrificed at indicated times afterbirth.

Bronchoalveolar lavage fluid (BALF) collection and VEGF analysis

Established protocols were followed to obtain BALF from the mice (Singh et al. 2009). Briefly, 10-wk old FA (control) and SS-exposed were anesthetized and killed by exsanguination. Before excision of the lungs, the trachea was surgically exposed, cannulated and, while the left lung lobe was tied off with a silk thread, the right lobe was lavaged twice with 0.8 ml sterile Ca^{2+}/Mg^{2+}-free PBS (pH 7.4). Aliquots were pooled from individual animals and centrifuged to separate cells and BALF. The concentration of VEGF in BALF was determined by ELISA using mouse-

specific VEGF ELISA kit (Biosource-Invitrogen, Camarillo, CA), according to the manufacturer's directions.

Tissue preparation, histochemistry, and immunostaining

After sacrificing the animals, lungs were removed, inflated and fixed at a constant hydrostatic pressure. All lungs were equally inflated on the same apparatus. Immediately after inflation, the trachea was ligated and the lungs were immersed in formalin for overnight fixation. The following day, the lungs were washed in PBS (3x), dehydrated in graded ethanol solutions, embedded in paraffin (Fritzell et al. 2009; Wang et al. 2001), and lung section (5 μm) staining were followed as described below.

BALF from the lungs of 10-wk and 8-month old animals were obtained as described above; however, after removal of BAL, right lungs from some animals were frozen instantaneously in liquid N_2 and stored at -80 °C. When needed these lungs were used for making lung homogenates and total RNA. Lungs from 7-day old mice were also kept frozen at -80 °C for Western blot analysis and total RNA isolation.

Immunohistochemical (IHC) staining for surfactant protein-B (SP-B)

For IHC staining lung section were deparaffinized by soaking in xylene for 10 min (2x), and then hydrated by 5-min treatment with decreasing concentrations of ethanol (100%, 95%, 70%, and 30%). Finally, the slides were rinsed three times with distilled water. The deparaffinized lung sections were immerged in 0.01 M sodium citrate buffer (pH 6.0) and heated in a microwave for 15 min at 90°C for antigen retrieval. Endogenous peroxidase activities were quenched with 3% H_2O_2 in methanol for 15 min at room temperature (RT). The tissue sections were blocked with 2% normal goat serum in PBS with 0.2% triton for 2 hours at RT, followed by overnight

incubation at 4°C with rabbit proSP-B antibody (1:1000 dilution; Cat# AB3430; Chemicon, CA). Sections were then washed (6x for 5 min each) in PBS with 0.2% Triton and incubated for 30 min at RT with goat anti-rabbit (1:200 dilution; Cat# BA-1000; Vector, Burlingame, CA) for 1 hour at RT. Slides were then incubated in Vectastain Elite Immunoperoxidase system at RT for 30 min followed by 3,3'-Diaminobenzidine Peroxidase Substrate for 5 min at RT. Finally the sections were counterstained with hematoxylin, and analyzed with a Nikon Eclipse E600W microscope (PXM1200F). Images of proximal and distal airways of the lungs were acquired using SP-B stained lung sections (Nikon Eclipse E600W microscope) to access SS-effect throughout the lung.

SPDEF (SAM pointed domain-containing Ets-like factor) staining

Deparaffinized and rehydrated lung section were followed by antigen retrieval and endogenous peroxidase quenching procedure as mentioned earlier. Slides were blocked with 10% goat serum for 2 hours at RT. The slideswere incubated overnight at 4°C with a guinea pig anti-mouse polyclonal SPDEF antibody (GP954, a generous gift from Dr. Jeffrey Whitsett, Cincinnati Children's Hospital) at 1:2500 dilution. Slides were washed (5x) with buffer and incubated at RT for 30 minutes with a 1:200 dilution of a goat anti-guinea pig biotinylated IgG (cat# BA-1000; Vector Lab, Inc. Burlingame, CA), washed in PBS and incubated for 30 minutes in the Immunoperoxidase Kit (cat# PK-6100; Vector) as per manufacturer's instructions. Washed slides (5x in buffer) were incubated for 2 minutes at RT in peroxidase substrate (cat#SK-4100; Vector) without nickel and counterstained with hematoxylin, dehydrated and examined under Nikon Eclipse E600W microscope (brown stains).

IHC staining for Clara cells

Deparaffinized lung sections were stained for Clara cell secretory protein (CCSP, also called CC10, CC16, or uteroglobin) according to the method described by Reynolds et al. 2000. Briefly, lung sections (5 µm) were incubated with rabbit anti-CCSP antibody (1:50000 dilution; cat # 957, Vector, CA) overnight at 4°C. The sections were then washed as before and incubated with goat anti-rabbit antibody (Cat # BA-1000, Vector, CA) at a dilution of 1:200 for 1 hour at RT. After washing, the sections were incubated in a Vectastain Elite Immunoperoxidase system at RT for 30 min followed by 3,3'-Diaminobenzidine Peroxidase Substrate for 10 min at RT, and counterstained with hematoxylin, and examined microscopically (Nikon Eclipse E600W). Proximal and distal lung airways images were acquired using CCSP stained lung sections (Nikon Eclipse E600W microscope) to access SS-effect throughout the lung

Immunofluorescence staining of epithelial surface cilia

To visualize ciliated Clara cells, lung sections were treated with anti-β-tubulin antibody (Rawlins et al. 2007; Tompkins et al. 2009). Deparaffinized lung section were permeabilized with 0.2% Triton X-100 in PBS for 5 min and stained with rabbit anti-β-tubulin antibody (Abcam) for 2 hours followed by Alexa 594-conjugated anti-rabbit antibody (Invitrogen) for 1 hour. Sections were washed 5 times with wash buffer (PBS with 0.025% Triton X-100) and treated with 10 µg/ml Hoechst dye (Invitrogen). Washed sections were mounted in Mowiol Mounting Media (Sigma-Aldrich). All the above steps were performed at RT. Image fluorescence was visualized by confocal microscopy (Zeiss LSM510 META confocal microscope). Acquired images were processed in Photoshop CS3.

CD34 and CD31 Immunofluorescence staining

To examine vascularization, lung sections were stained with anti-mouse CD34 antibody (1:2000 dilution, Cat#:119301, BioLegend, San Diego, CA), and rabbit anti-CD31 (1: 2000 dilution, Cat #: ab28364, Abcam, Cambridge, MA) respectively using standard procedures. Lung sections were evaluated for expression of CD34 and CD31 by immunofluorescence. Briefly, deparaffinized, and hydrated lung sections were treated with 0.1M citrate buffer (pH 6.0). Lung sections were blocked and incubated with anti-rat Alexa 555 (Life Tech., NY), and anti-rabbit DyLight 549 (Jackson Immunoresearch Labs, West Grove, PA) for CD34 and CD31 stained lung slides, respectively. Stained sections were mounted with 4', 6-diamidino-2-phenylindole (DAPI) containing Fluormount-G^{TM} (Southern Biotech, Birmingham, AL) for nuclear staining (blue fluorescence). Fluorescently-labeled sections were analyzed with a Nikon Eclipse E600W microscope with a digital camera (PXM1200F) using Axioplan 2 imaging system (Carl Zeiss Microimaging Inc., Thornwood, NY) and acquisition software Slidebook^{TM} 5 (Intelligent Imaging Innovations, Inc. Denver, CO).

Trichrome Staining

To determine lung fibrosis, lung sections were stained with Masson's Trichrome (Sigma-Aldrich) to reveal collagen (see Supplemental Material, Figure S2) following standard protocol.

Western blot analysis

CD34 were quantitated by Western blotting of the lung homogenates as described previously (Singh et al. 2003; Singh et al. 2011). Briefly, lung tissues were homogenized in RIPA buffer (20 mM Tris, 150 mM NaCl, 20 mM β-glyceryl-phosphate, 1% Triton-X, 10 mM NaF, 5 mM EDTA, 1 mM Na_3VO_4 and protease inhibitors (1 mM PMSF, and 1 μg/ml each of aprotinin,

antipain, and leupeptin) at 4°C. Total protein content of the extracts was determined by the BCA Protein Assay Kit (Pierce, Rockford, IL) according to the manufacturer's directions. Lung homogenates proteins were resolved by SDS-PAGE on 10% precast gels (Invitrogen) and transferred to nitrocellulose membranes (Bio-Rad, Hercules, CA). The blots were probed with anti-CD34 antibody (MEC14.7, Abcam), and the antibody-bound proteins on the blot were detected with enhanced chemiluminescence (Amersham Biosciences, UK) on an X-ray film.

Supplemental Material Figure S1

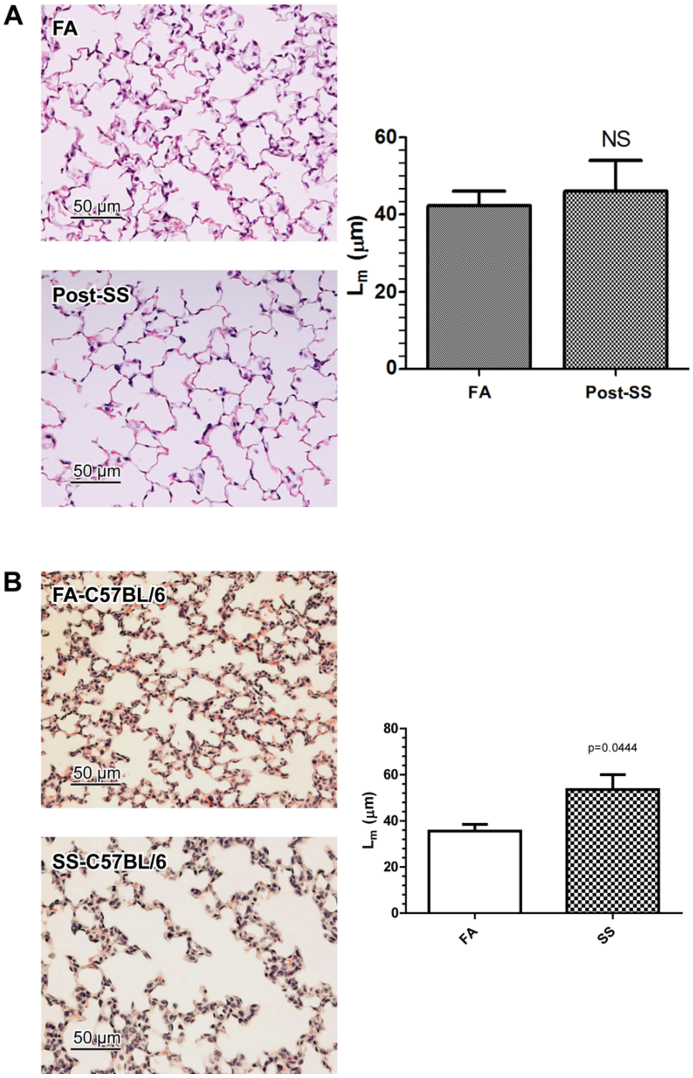

Supplemental Material Figure S1. (A) Postnatal exposure to SS does not affect alveolarization. Representative lung (H&E stained) sections (40x magnification) from 10-week FA (left, top: control animal) and SS (left, bottom: gestationally SS-exposed). L_m (right bar graph, Fig. S1A)**. (B) C57BL/6 mice are also sensitive to gestational exposure to SS.** Representative C57BL/6 mice lung (H&E stained) sections (40x) of 7-day old mice (left, top: control animal) and SS (left, bottom: gestationally SS-exposed). L_m (right bar graph, Fig. S1B). Data are mean plus/minus SD; $p \leq 0.05$ significant**;** NS = not significant (n = 5). FA: filtered air; SS: gestationally exposed to secondhand cigarette smoke.

Supplemental Material Figure S2

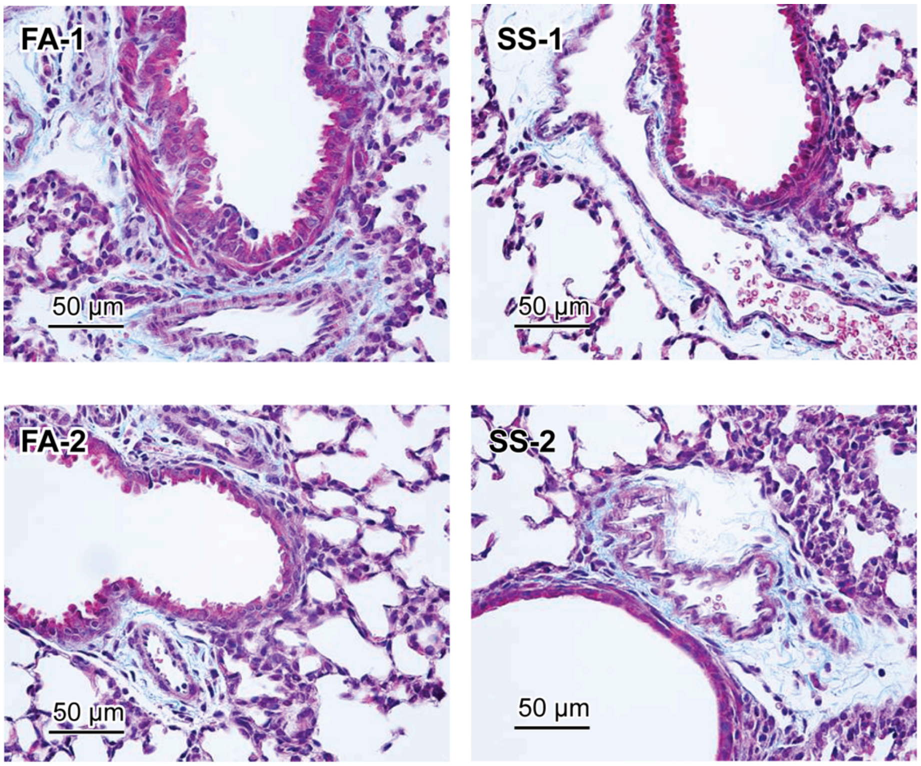

Supplemental Material Figure S2. Lung collagen is unaffected by gestational exposure to SS. Lungs section from 7-day old mice were stained with Masson's Trichrome to reveal collagen. FA (left panels), and SS (right panels). The blue staining represents collagen deposition (40x magnification). FA: filtered air; SS: gestationally exposed to secondhand cigarette smoke, FA-1/SS-1 and FA-2/SS-2 indicate separate animal identity.

Supplemental Material Figure S3

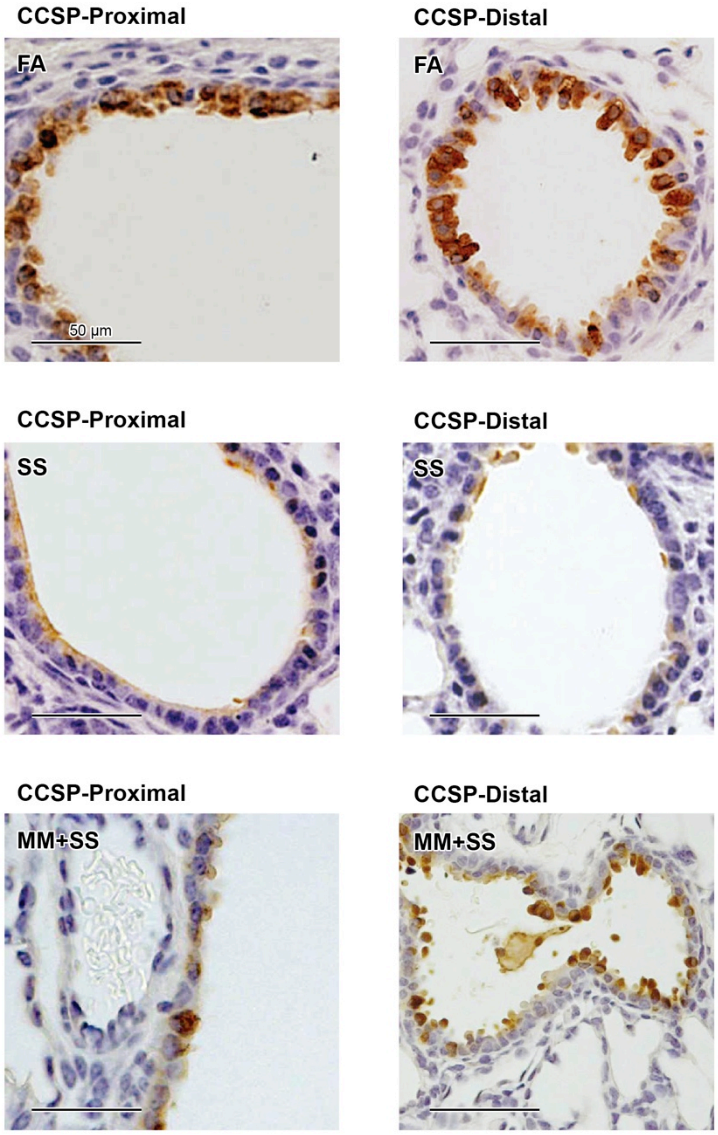

Supplemental Material Figure S3. Gestational SS-exposure affects CCSP throughout the airways and reversed by mecamylamine (MM). Representative lung sections (40x magnification) of 7-day old mice stained with anti-CCSP antibody as described in Materials and Methods section. FA = filtered air (control); SS = gestational secondhand cigarette smoke, and MM+SS = MM-treated and gestationally SS-exposed mice.

Supplemental Material Figure S4

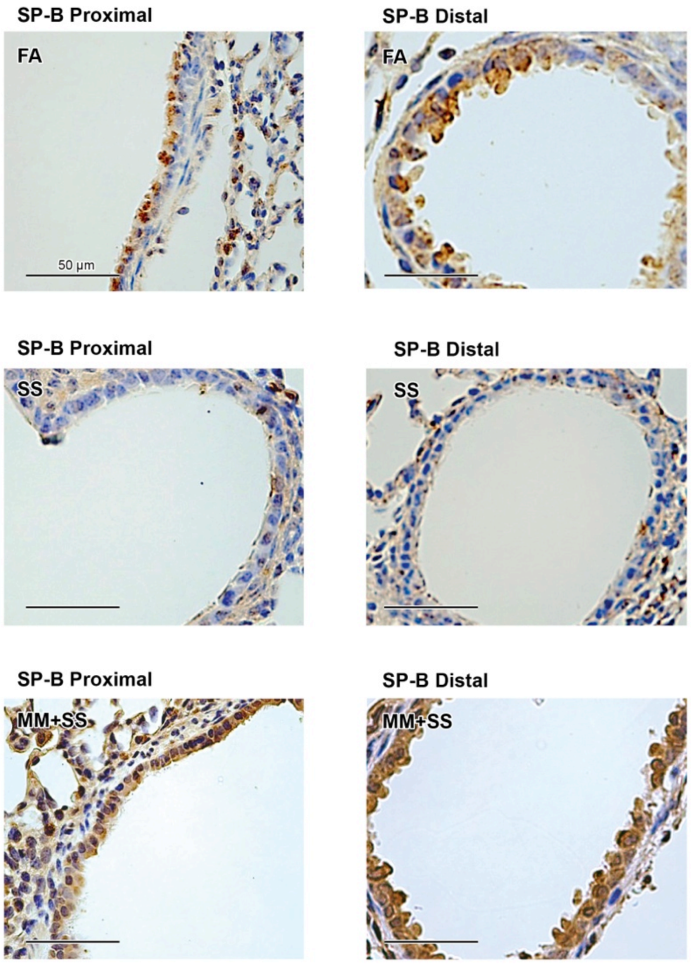

Supplemental Material Figure S4: Gestational SS-exposure affects SP-B throughout the airways and reversed by mecamylamine (MM). Representative lung sections (40x magnification) of 7-day old mice stained with anti-SP-B antibody as described in Materials and Methods. FA = filtered air (control); SS = gestational secondhand cigarette smoke, and MM+SS = MM-treated and gestationally SS-exposed mice.

Supplemental Material Figure 5

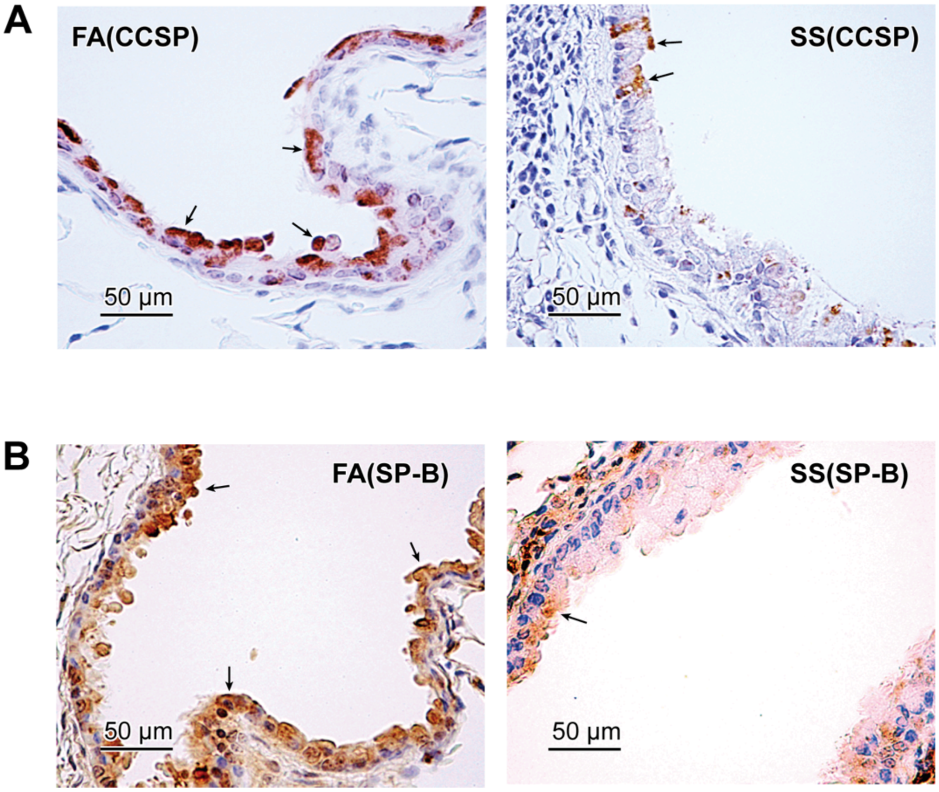

Supplemental Material Figure S5. CCSP and SP-B are reduced in SS-exposed lungs at 10 wk afterbirth. Representative lung section (40x magnification) of 10-week old mice (gestationally exposed to either FA or SS) stained with anti-CCSP antibody (A) or stained with anti-SP-B antibody (B) as described in Materials and Methods. FA: filtered air; SS: gestationally exposed to secondhand cigarette smoke, → directs toward positive cells in the airway (n =5).

References

Fritzell JA, Jr., Mao Q, Gundavarapu S, Pasquariello T, Aliotta JM, Ayala A, et al. 2009. Fate and effects of adult bone marrow cells in lungs of normoxic and hyperoxic newborn mice. Am J Respir Cell Mol Biol. 40:575-587.

Knudsen L, Weibel ER, Gundersen HJ, Weinstein FV, Ochs M. 2010. Assessment of air space size characteristics by intercept (chord) measurement: an accurate and efficient stereological approach. J Appl Physiol 108:412-421.

Rawlins EL, Ostrowski LE, Randell SH, Hogan BL. 2007. Lung development and repair: contribution of the ciliated lineage. Proc Natl Acad Sci USA 104:410-417.

Reynolds SD, Giangreco A, Power JH, Stripp BR. 2000. Neuroepithelial bodies of pulmonary airways serve as a reservoir of progenitor cells capable of epithelial regeneration. Am. J. Pathol. 156:269-278.

Singh SP, Barrett EG, Kalra R, Razani-Boroujerdi S, Langley RJ. et al. 2003. Prenatal cigarette smoke decreases lung cAMP and increases airway hyperresponsiveness. Am J Respir Crit Care Med. 168:342-47.

Singh SP, Mishra NC, Rir-Sima-Ah J, Campen M, Kurup V, Razani-Boroujerdi S, et al. 2009. Maternal exposure to secondhand cigarette smoke primes the lung for induction of phosphodiesterase-4D5 isozyme and exacerbated Th2 responses: rolipram attenuates the airway hyperreactivity and muscarinic receptor expression but not lung inflammation and atopy. J Immunol 183:2115-2121.

Singh SP, Gundavarapu S, Pena-Philippides JC, Rir-Sima-Ah J, Mishra NC, Wilder JA, et al 2011. Prenatal secondhand cigarette smoke promotes Th2 polarization and impairs goblet cell differentiation and airway mucus formation. J Immunol 187:4542-4552.

Tompkins DH, Besnard V, Lange AW, Wert SE, Keiser AR, Smith AN, et al 2009. Sox2 is required for maintenance and differentiation of bronchiolar Clara, ciliated, and goblet cells. PLoS One 4:e8248.

Wang SZ, Rosenberger CL, Espindola TM, Barrett EG, Tesfaigzi Y, Bice DE, et al. 2001. CCSP modulates airway dysfunction and host responses in an Ova-challenged mouse model. Am J Physiol 281:L1303-L1311.
